# The Treatment of Infected Wounds of the Extremities

**Published:** 1904-12

**Authors:** Marshall Clinton

**Affiliations:** Buffalo, N. Y., Attending Surgeon Buffalo Hospital Sisters of Charity and Erie County Hospital; Instructor of Clinical Surgery, University of Buffalo, etc.


### The Treatment of Infected Wounds of the Extremities.

By MARSHALL CLINTON, M. D., Buffalo, N. Y.,

Attending Surgeon Buffalo Hospital Sisters of Charity and Erie County Hospital; Instructor of Clinical Surgery, University of Buffalo, etc.

THE treatment of infected wounds of the extremities, to be rational, should be based upon a proper understanding of the agencies that cause infection. A study, in a general way, of all the agents possible of producing an infected wound show us that the temperature at which these agents grow best is the normal body temperature. Experimentation with the pyogenic organisms has shown that a slight increase in the temperature above the optimum is sufficient materially to modify their growth, in many cases to stop the growth, and in others actually destroy them.

It has been known for a long time that a culture of the streptococcus pyogenes will die after forty-eight hours if the temperature of the culture is kept at 106° F. A slight rise of two or more degrees above the optimum temperature of the pus organisms will materially modify their growth and virulency. With all organisms the usual method of producing attenuated organisms in culture is to grow them at a temperature slightly above their optimum temperature. The thermal death point of all the pus organisms is given for short periods in minutes, but the lowest temperature that will kill these organisms after forty-eight or more hours is known only in the case of the streptococcus pyogenes. We know that the pneumococcus ceases to grow at 104° F.—a fact of clinical interest to medical men.

Acting upon the suggestion that a rise in temperature attenuates and destroys the pyogenic bacteria, a series of experiments were carried out to try and determine how high the temperature of the tissues of an extremity could be raised without harm.

A series of twenty-five experiments were carried out in the following manner: a hot air machine, of the small type in common use, was especially constructed with a funnel, shaped from the top of the machine leading into the center. The funnel was 4 inches deep, 4 inches across the top and 1 inch at the bottom. A thermometer was inserted into a small sinus to a depth of from ½ to 2 inches, and around the thermometer, and for a distance of ½ inch on every side was wound asbestos packing. The base of the funnel was pressed down tightly on to the asbestos packing, so that the thermometer as it emerged from the tissues and the packing was entirely outside the machine. The heat was then started with a Bunsen flame and the extremity heated under the machine, except just over the sinus and packing. It is found that the temperature of the hot air machine is tolerated by bare skin up to about 235° F. before there is much discomfort. The tissues in the extremity are raised in temperature to a point indicated by the buried and insulated thermometer. When a sinus in an extremity was used a temperature of from 102° to 106° F. was reached in the tissues, depending on the degree of heat obtained in the heater. It takes about fifteen minutes for the tissues to rise in temperature after the thermometer in the heater has gone up. The heat is retained in the tissues for about fifteen minutes after the hot air machine is removed. The temperature in the tissues will remain up as long as the hot air machine is left on.

For the past four years this method of treating infected wounds of the extremities by hot air has been employed and in every case the clinical results bear out the thought of the parallel behavior of organisms in culture media at the same temperature. No antiseptics are used on the wounds; they are simply covered with a sterile light dressing of gauze, to permit the heat to gain access. If a patient with a badly infected extremity who is being treated by this method leaves his hand out of the hot air for a few hours there will be a return of that stinging pain and a gradual rise in his temperature, showing the renewed growth on organisms that have been inhibited. In cases such as pyoarthrosis of the knee, which are always excessively sensitive and accompanied by much septic fever, it was found that this method of treatment eliminated a large share of these symptoms. When a hand or foot is badly injured with promise of much sloughing of soft tissue, this procedure gives better results than the macerating hot bath. The value of a hot bath is simply the degree of heat imparted to the tissues and here we may use a method that will convey as much or more heat steadily, without the drawbacks of the water maceration. Large areas of dead tissue may slough off without the development of the cellulitis, so often seen above the dead tissue. In cases of bone tuberculosis, with discharging sinuses, the septic element in the case as such may be materially modified by the continuous use of hot air.

The usual method of using hot air has been to apply it for an hour or more in rheumatic and traumatic conditions, but to be efficacious in septic conditions it should be used for from 12 to 20 hours out of each 24 hours.

This procedure is not recommended to the exclusion of such proper surgical principles as drainage, removal of dirt from a wound, relief of undue tension and the like, yet, combined with proper surgical procedures, it will be found most useful.

466 Franklin Street.

---

FOR CYSTITIS.

℞ Urotropin .................................... gr. vijss.
Sodii phosphatis (acid) ......................... gr. xxx.

M.—Ft. tales chart. No. xxx. Sig.—One powder dissolved in a glass of Vichy water three or four times daily.

---

TONSILLITIS.

℞ Sod. benzoat .................................... ℨi.

Ext. cascara sagrad .................................. fl ℨi.

Tongaline .................................... q. s. ad ℥vi.

M. Sig.—A teaspoonful every two to four hours.

